# CIC::DUX4 Sarcoma With Preferentially Expressed Antigen in Melanoma (PRAME) Immunopositivity

**DOI:** 10.7759/cureus.85154

**Published:** 2025-05-31

**Authors:** John Grove, Rana Naous

**Affiliations:** 1 Pathology and Laboratory Medicine, University of Pittsburgh Medical Center, Pittsburgh, USA; 2 Pathology, University of Pittsburgh Medical Center, Pittsburgh, USA

**Keywords:** cic, dux4, fusion, prame, sarcoma

## Abstract

*CIC*-rearranged sarcomas are rare, high-grade, undifferentiated, small round cell sarcomas of bone and soft tissue classified by gene fusions involving the *CIC* gene with other gene partners, most commonly the *DUX4* gene. These tumors tend to affect a wide age range, with a predilection for adult males, with the most common anatomical location being the deep soft tissues of the limbs or trunk. *CIC*-rearranged sarcomas have proven not only to be challenging to diagnose but also to treat due to their high resistance to conventional therapies. Preferentially expressed antigen in melanoma (PRAME) is an immunohistochemical stain that was developed primarily for the diagnosis of melanoma and has been shown to also be expressed in other epithelial and mesenchymal tumors. To date, PRAME immunopositivity has not been reported in *CIC*-rearranged sarcomas. Here, we report a 51-year-old male with a large gluteal undifferentiated small round cell sarcoma and bilateral lung metastases, whereby RNA sequencing confirmed the tumor as *CIC::DUX4* sarcoma. Interestingly, the sarcoma demonstrated very strong PRAME immunohistochemical positivity, a diagnostic finding that has not yet been reported on *CIC::DUX4* sarcomas and has potential use as a beneficial tool in the workup of a diagnostically challenging disease.

## Introduction

Capicua transcriptional repressor (*CIC*)-rearranged sarcomas are undifferentiated, small, round cell sarcomas that are clinically aggressive and demonstrate poor response to chemotherapy [1]. *CIC-DUX4* rearranged sarcoma comprises the vast majority (95%) of *CIC*-rearranged sarcoma cases [2]. The *CIC* gene encodes a high mobility group box transcription factor that becomes fused to the *DUX4 *gene and results in a chromosomal translocation including t(4;19)(q35;q13) or t(10;19)(q26;q13) [2,3]. Other *CIC*-fusion partner genes include* FOXO4, LEUTX, NUTM1*, and *NUTM2A *[1]. The age range for CIC-rearranged sarcomas varies widely, with cases reported from ages 6 to 81 years old [4]. The majority of these sarcomas are found in the deep soft tissue of the limbs or trunk, with reported involvement of other sites such as the bone, head and neck, and viscera [1]. *CIC*-rearranged sarcomas tend to manifest clinically as painful, rapidly growing soft tissue masses, but at times they can be painless and can even go unnoticed until there is metastatic spread [1]. Like most neoplasms, the diagnostic workup of a CIC-rearranged sarcoma begins with morphologic and immunohistochemical evaluation of the resected specimen. *CIC*-rearranged sarcomas are diagnostically challenging, as they are undifferentiated sarcomas with a predominant round cell phenotype that can mimic many other tumors, including Ewing sarcoma. While *CIC*-rearranged sarcomas tend to be positive for CD99, WT1, ETV4, and DUX4, these stains are fairly nonspecific and are not sensitive enough to be used alone, thus, there remains a need for specialized genetic testing such as RNA sequencing or RT-PCR for confirmatory diagnosis. Given the rarity of these tumors, lack of awareness can be a contributing factor to the diagnostic complexity of *CIC*-rearranged sarcomas.

Preferentially expressed antigen in melanoma (PRAME), a marker that was developed primarily for the diagnosis of melanoma, is a nuclear receptor, transcriptional regulator, and a member of the cancer testis antigen family of proteins that regulates cell differentiation, growth, and apoptosis. In the context of melanomas, PRAME has a high sensitivity (>90%) and specificity. However, PRAME has been shown to be positive in tumors other than melanoma, including many epithelial and non-epithelial tumors and a few sarcomas, leading to variable sensitivity and sensitivity in that regard. In the case presented, we call attention to the strong immunopositivity for the PRAME immunostain in *CIC::DUX4* sarcoma. Such a novel finding could serve as a useful diagnostic tool to place *CIC::DUX4* sarcomas higher on the differential diagnosis in soft tissue tumors and increase the diagnostic rate of this disease [5].

## Case presentation

Clinical presentation

A 51-year-old male with a history of deep vein thrombosis (DVT) presented to the hospital for persistent right hip and buttock pain, sciatica, and decreased right lower extremity function. Three months prior, the patient noticed pain and localized swelling in the right buttock after experiencing a ground-level fall.

Imaging studies

**Figure 1:**
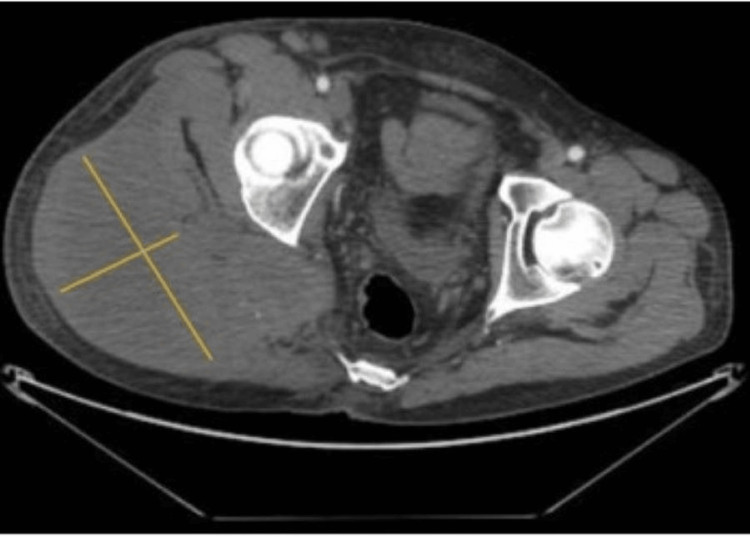
CT abdomen CT abdomen showing a large, irregular, necrotic right gluteal mass

Laboratory tests were non-contributory. CT angiography of the abdomen and pelvis demonstrated a large, irregular, necrotic mass invading the right gluteal muscles and extending through the right sciatic notch measuring 17 x 15 x 13 cm (Figure 1), as well as satellite necrotic masses involving the right greater sciatic notch and right hemipelvis, the largest of which measured 5.1 x 4.7 cm.

**Figure 2:**
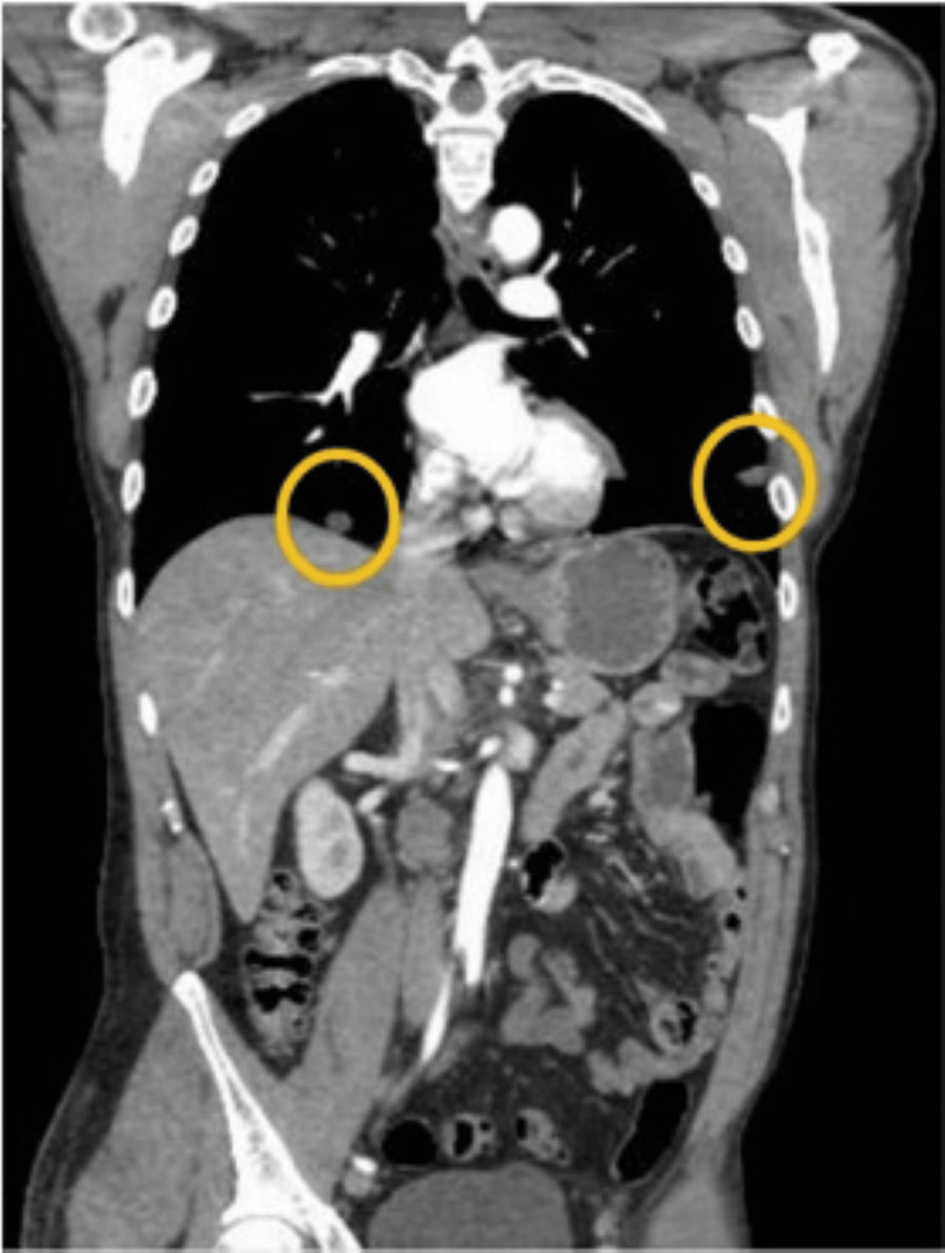
CT chest CT angiography of the chest demonstrating bilateral pulmonary metastases

The imaging study was also positive for focal tumor invasion of the right common iliac vein with an adjacent deep vein thrombosis (DVT) and a metastatic necrotic retrocaval lymph node measuring 2.3 cm in the greatest dimension. CT angiography of the chest revealed multiple bilateral metastatic pulmonary nodules, the largest of which measured 2.4 cm (Figure 2).

Histologic findings

**Figure 3:**
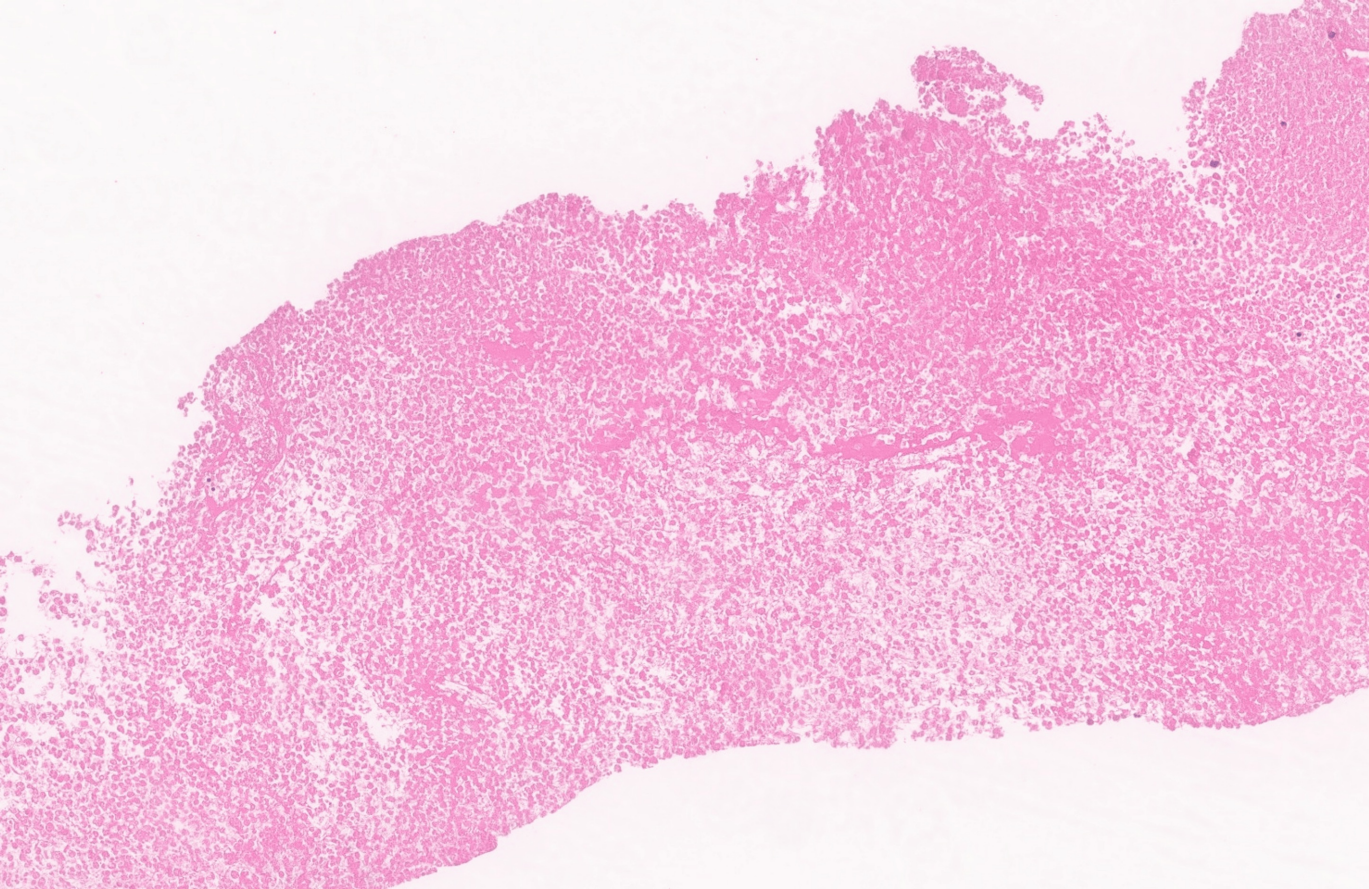
Gluteal mass biopsy Gluteal mass biopsy demonstrating a predominantly necrotic sample with no viable tumor cells (H&E; 10x)

An ultrasound-guided biopsy of the right gluteal mass was performed and was predominantly necrotic (Figure 3).

**Figure 4:**
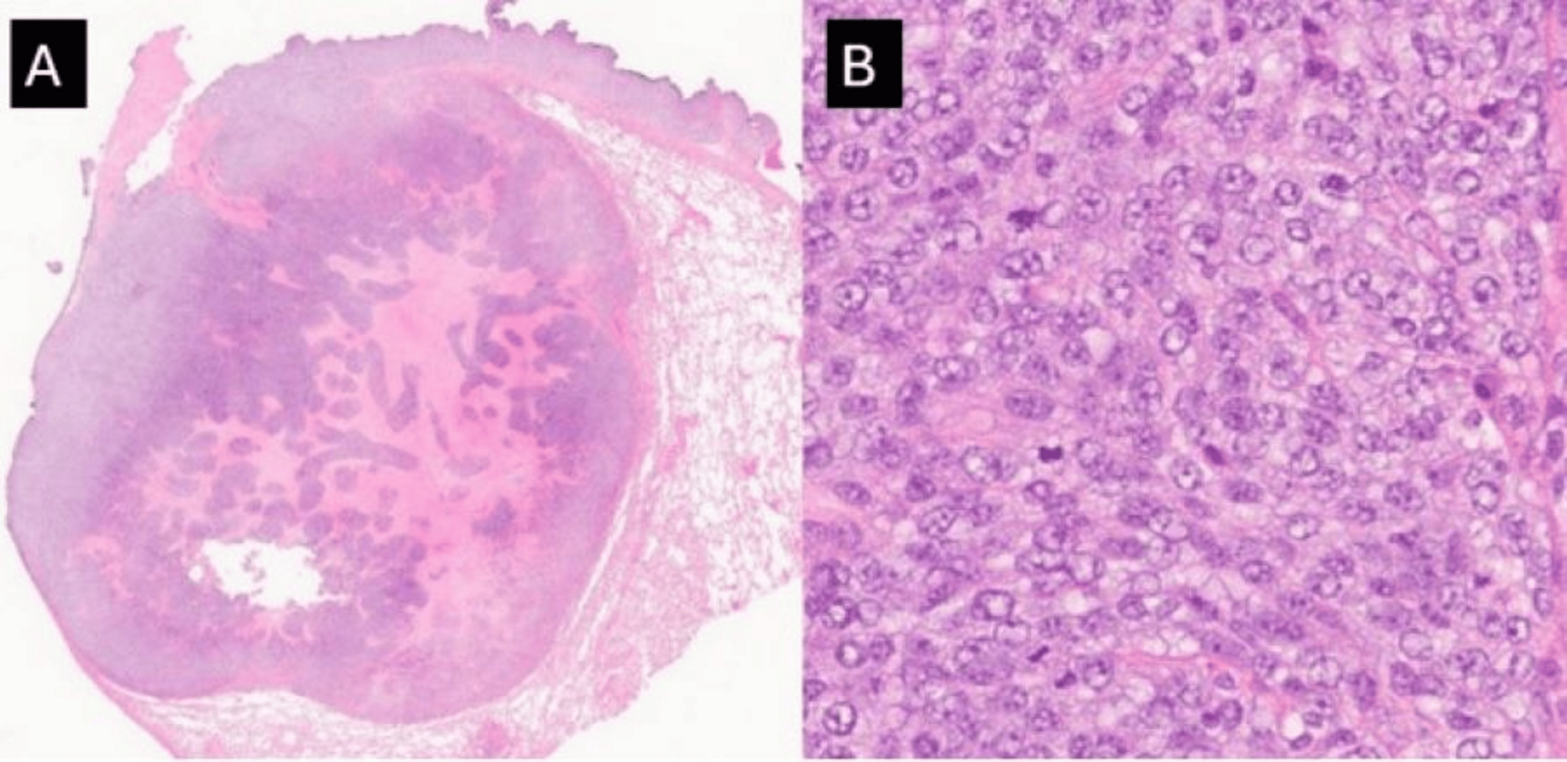
Metastatic CIC::DUX4 sarcoma A: Low-power magnification demonstrating the well-circumscribed nodular nature of the tumor metastatic deposit within the lung wedge resection (H&E;2x), B: Higher power magnification of the tumor showing cellular sheets of malignant round cells with a moderate nuclear-to-cytoplasmic (N:C) ratio, vesicular chromatin, prominent nucleoli and pale frothy cytoplasm (H&E, 100x).

Subsequently, the patient underwent a left lower lobe lung wedge resection that contained three separate tumor nodules ranging in size from 0.9 to 2.3 cm. Microscopic evaluation of the lung nodules revealed a similar morphology and consisted of cellular sheets and nests of malignant round cells with a moderate nuclear-to-cytoplasmic (N:C) ratio, vesicular chromatin, prominent nucleoli, and pale frothy cytoplasm with associated necrosis (Figure 4).

**Figure 5:**
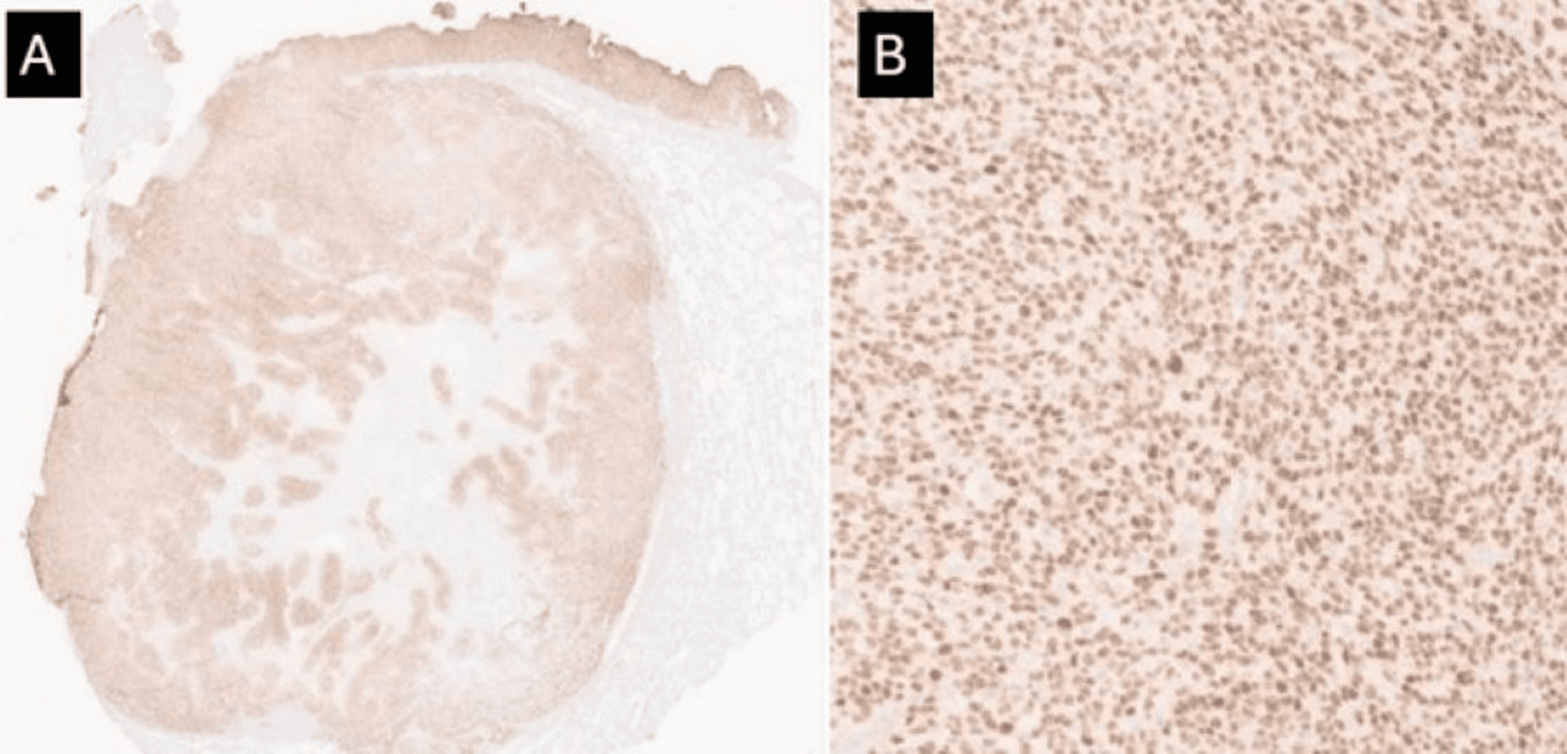
PRAME immunostain Low (A) and high (B) power magnification demonstrating diffuse strong staining for PRAME within the tumor cells (2x, 100x). PRAME: preferentially expressed antigen in melanoma

**Figure 6:**
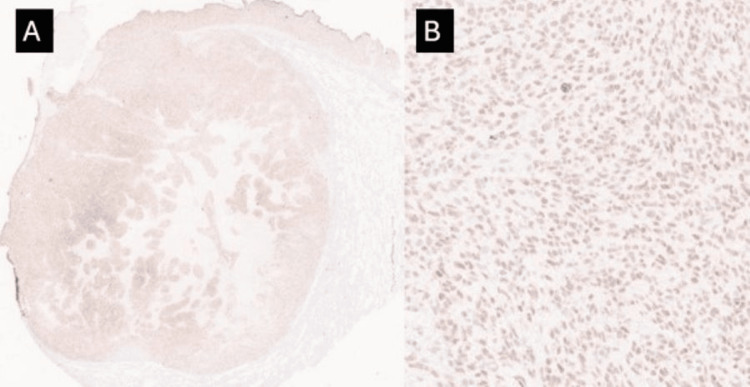
WT1 immunostain Low (A) and high (B) power magnification demonstrating diffuse weak to moderate staining for WT1 within the tumor cells (2x, 100x)

The tumor extended through the pleural surface. Immunohistochemical stains were diffusely and strongly positive for PRAME (Figure 5) while WT1 exhibited diffuse weak to moderate staining (Figure 6).

All other immunostains including S100, SOX10, cytokeratin AE1/AE3, desmin, myogenin, MyoD1, and CD45 were negative and non-contributory.

Molecular studies

Targeted next-generation sequencing (NGS) analysis of 161 genes was performed using the institutional Oncomine™ Comprehensive Assay v5.12 using the Ion Torrent™ NGS platform (ThermoFisher Scientific, Waltham, MA, USA) according to the manufacturer’s instructions. Oncomine studies were negative for DNA mutations, gene fusions, and copy number alterations. Whole transcriptome exome sequencing (RNA Seq) was performed and exhibited *CIC::DUX4* gene fusion (*5’ CIC* breakpoint corresponding to chr19:42799157 and *3’ DUX4* breakpoint corresponding to chr4:191006158).

## Discussion

*CIC*-rearranged sarcomas are undifferentiated, small, round cell sarcomas with high-grade, round cell morphology that harbor genetic fusions involving the *CIC* gene [1]. While multiple *CIC* gene fusions have been documented in the medical literature, the vast majority of *CIC*-rearranged sarcomas harbor the *CIC::DUX4* gene fusion.

*CIC::DUX4* sarcomas can arise in persons of any age group, but most commonly present in young adults between the ages of 25 and 35, with rare cases being reported in young children and older adults. They tend to affect males slightly more than females [4,6]. These tumors are often localized to the deep soft tissues in the limbs or trunk and vary in clinical presentation, with some masses causing significant pain and disability while other cases being relatively asymptomatic [6]. In our case, the patient had significant pain due to local invasion, resulting in sciatica and diminished lower extremity function.

Morphologically, *CIC::DUX4* sarcomas appear undifferentiated and commonly display a population of monomorphic, predominantly round cells. According to the WHO, epithelioid or spindle cell components may be present as well. Due to its high-grade nature, frequent mitoses and necrosis are commonly encountered [2]. *CIC::DUX4* sarcomas have a high rate of distant metastases, especially to the lungs, as also seen in the case above [7].

The differential diagnosis for undifferentiated small round cells involving the deep soft tissue is quite broad, with some entities to consider, including round cell sarcomas with* EWSR1* fusions, round cell sarcomas with *BCOR* alterations, and small round cell sarcomas, not otherwise specified (NOS). Round cell sarcomas with *EWSR1 *fusions include Ewing sarcoma and round cell sarcomas with *EWSR1*-non-*ETS* fusions such as *EWSR1::NFATC2* and *EWSR1::PATZ1* sarcomas. The distinction between *CIC*-rearranged sarcomas and Ewing sarcoma is important, as they share many similar morphologic characteristics; however, *CIC::DUX4* sarcomas have a much poorer prognosis [4]. Fluorescence in situ hybridization (FISH) studies for *EWSR1* gene rearrangement or whole transcriptome exome sequencing can help assist in excluding an *EWSR1*-rearranged sarcoma, round cell sarcomas with *BCOR* alterations, and small round cell sarcomas, NOS, respectively. *CIC::DUX4* sarcomas are diagnostically challenging, mainly due to a lack of awareness and a lack of a specific immunostaining pattern [2]. Immunohistochemical staining for *CIC::DUX4* sarcomas is most notably positive for WT1 and CD99 [8]. While WT1 and CD99 staining are sensitive, they are not specific for CIC-rearranged sarcomas. Ewing sarcomas and *CIC::DUX4* sarcomas are both CD99 positive; however, Ewing sarcomas tend to have a more distinct, membranous CD99 staining pattern [8]. In this case, the PRAME immunostain showed strong and diffuse nuclear positivity within the tumor (Figure 5). While PRAME was developed to distinguish melanoma from benign nevocytes, recent studies have shown that PRAME can stain numerous types of epithelial and non-epithelial tumors [5], including synovial sarcomas, myxoid liposarcomas, neuroblastomas, malignant peripheral nerve sheath tumors, and a subset of carcinomas. The strong staining in our case underscores a new potential use of the PRAME immunostain as a cost-effective method to effectively screen for *CIC::DUX4* sarcomas in the initial workup of an undifferentiated round cell soft tissue sarcoma.

*CIC*-rearranged sarcomas tend to have a poor prognosis and a high incidence of metastatic disease at the time of presentation [9]. As in our case, the patient was found to have bilateral pulmonary metastases on imaging. A major contributing factor to the poor prognosis is their insensitivity to many chemotherapy regimens [9]. This can be contrasted to Ewing sarcomas, which tend to respond much better to chemotherapy treatment regimens [9].

## Conclusions

*CIC*-rearranged sarcomas are rare soft tissue tumors that are clinically and morphologically aggressive. These tumors tend to present at a high stage and can be difficult to diagnose and treat. Here, we report a case of a 51-year-old male who presented after several months of pain related to a gluteal tumor with distant metastases. The initial workup of this undifferentiated tumor demonstrated strong PRAME immunopositivity in a tumor ultimately deemed to be a *CIC::DUX4* sarcoma via whole transcriptome exome sequencing. PRAME positivity in *CIC::DUX4* sarcomas has not been documented in the current medical literature; we hope that it will serve as a useful diagnostic tool in the workup of these challenging tumors and may further assist our understanding of their underlying pathogenesis. More cases are needed to further evaluate the utility of the PRAME immunostain in undifferentiated round cell soft tissue sarcomas. 
